# A CSF-1R Ig4-5 Domain-Targeting Antibody for the Treatment of Idiopathic Pulmonary Fibrosis

**DOI:** 10.3390/antib15040076

**Published:** 2026-08-11

**Authors:** Wusong Luo, Zhe Shao, Tao Wang, Kenghoe Lok, Rongjing Zhang, Yao Li

**Affiliations:** 1 State Key Laboratory of Genetics and Development of Complex Phenotypes, Shanghai Engineering Research Center of Industrial Microorganisms, School of Life Sciences, Fudan University, Shanghai 200438, China; luowusong@163.com; 2 Dragon Boat Biopharmaceutical Co., Ltd., Shanghai 201203, China

**Keywords:** CSF-1R, antibody, IPF, targeted therapy, macrophage

## Abstract

Background: Idiopathic pulmonary fibrosis (IPF) is closely associated with fibroblast proliferation, macrophage polarization, and the accumulation of extracellular matrix (ECM). Targeting CSF-1R can rebalance macrophages, reduce ECM deposition, and block pro-fibrotic signaling. Methods: A Fab fragment targeting CSF-1R was screened via phage display. After sequence optimization, it was constructed into an IgG1 antibody (BC006). In vitro activity, domain binding, and signaling blockade were investigated, and in vitro safety indicators were evaluated. Efficacy was assessed using an induced IPF organoid-on-a-chip and bleomycin-induced mouse model. In vivo safety evaluation was conducted in cynomolgus monkeys. Results: BC006 showed an EC_50_ value of 226 ± 57 nM and a KD of 30 ± 2 nM. It specifically bound to the Ig4-5 domain of CSF-1R by inhibiting receptor dimerization without blocking ligand binding. It could dose-dependently inhibit the differentiation of monocytes into M2 macrophages and exert anti-IPF effects. In the induced IPF organoid-on-a-chip model, BC006 maintained lung barrier function and decreased α-SMA and Collagen I. It also improved lung function and attenuated the degree of fibrosis in the mouse model. Moreover, BC006 had no ADCC, CDC, cytokine release, or hemagglutination, and demonstrated favorable safety profiles in cynomolgus monkeys. Conclusions: BC006 is a novel anti-fibrosis antibody specifically targeting the CSF-1R Ig4-5 domain and offers a new therapeutic strategy for IPF.

## 1. Introduction

The CSF-1/CSF-1R signaling transduction regulates the survival, proliferation, differentiation, and migration of the monocyte–macrophage lineage [1]. Overexpression of CSF-1R and its ligands leads to abnormal activation of this signaling pathway [2]. CSF-1R antibodies specifically target CSF-1R molecules on the surface of macrophages in situ. By blocking the CSF-1R signaling pathway, they inhibit the differentiation of monocytes into M2-type macrophages, reduce the number of immunosuppressive macrophages, modulate the immune microenvironment, and exert effects such as anti-fibrotic and anti-tumor activities [3,4]. The purpose of this study was to conduct preclinical efficacy research on BC006 for IPF, based on the aforementioned mechanism of action of the signaling pathway.

IPF is a deadly lung disease with currently limited treatment options [5]. Globally, only three drugs have been approved for the treatment of IPF: Pirfenidone, Nintedanib, and Nerandomilast. Pirfenidone and Nintedanib were approved by FDA in 2014, and Nerandomilast (PDE4B inhibitor) from Boehringer Ingelheim in October 2025 [6,7]. Among the IPF drugs under clinical development, FibroGen’s Pamrevlumab and Roche’s Zinpentraxin Alfa have entered Phase III clinical trials [8,9]. Other molecules currently being investigated include TKI, PDE4B, CSF-1R, CTGF, PTX-2, JAK-STAT, and LPA [10,11,12,13,14,15].

IPF is typically characterized by fibroblast proliferation and massive accumulation of extracellular matrix, accompanied by tissue structure destruction and inflammatory damage [16]. After normal alveolar tissue is damaged, abnormal repair results in end-stage changes in a large category of lung diseases with abnormal structure [17]. Idiopathic pulmonary fibrosis is a life-threatening disease characterized by progressive and irreversible pulmonary fibrosis [18]. The median survival time is only 3 years. Although the basic mechanism of the pathogenesis of IPF is poorly understood, its hallmark pathological features include inflammation, excessive proliferation of fibroblasts, and abnormal deposition of extracellular matrix [19]. In this study, we aim to explore the mechanism of action of CSF-1R antibody in exerting anti-fibrotic efficacy through in vitro and in vivo experiments, and to investigate the anti-pulmonary fibrosis efficacy of BC006 by constructing organoid-on-a-chip and mice models.

## 2. Materials and Methods

Binding Activity Assay: The binding activity was determined using the enzyme-linked immunosorbent assay (Molecular Devices, San Jose, CA, USA). Human CSF-1R antigen (Sino Biological, Beijing, China) was coated on the plate, followed by the addition of serially diluted BC006. After incubation, horseradish peroxidase (HRP)-labeled anti-human IgG Fc secondary antibody (Sigma, St. Louis, MO, USA) was added. Color development was achieved through an enzymatic reaction. A four-parameter model was used to fit and analyze the antibody concentration and corresponding absorbance values, and the binding activity of the sample relative to the reference standard was calculated.

Affinity Assay: Affinity was evaluated using the ForteBio platform (Version 12.0, Sartorius, Fremont, CA, USA) based on bio-layer interferometry (BLI) technology. A Protein A sensor was used, with 5 μg/mL BC006 as the immobilized ligand and serially diluted CSF-1R as the analyte. The kinetic detection program was set as follows: loading for 30 s, association for 150 s, dissociation for 180 s, and regeneration for 5 s. The sample concentration and binding response values were fitted using a 1:1 binding model, followed by global analysis to calculate the KD value of the sample. The assay was considered valid when R^2^ ≥ 0.90.

Ability to Bind to Different Domains: ELISA was used to detect the ability of BC006 to bind to different domains of human CSF-1R, in order to determine the binding domain of BC006 on CSF-1R. Different domain antigens of human CSF-1R (Ig1-3: Met1-Ser290; Ig4-5: Ala299-Glu512) were coated in 96-well plates (Corning, Corning, NY, USA). Serially diluted BC006 and enzyme-conjugated anti-human Fc secondary antibody were added sequentially. After substrate color development, a four-parameter curve was fitted with the logarithm of antibody concentration as the abscissa and the OD450 value (reflecting the amount of antigen–antibody binding) as the ordinate. The binding activity of the sample relative to the reference standard was calculated.

Effect on CSF-1/IL-34-CSF-1R Interaction: (1) Competitive ELISA. Human CSF-1R antigen was coated in 96-well plates. A fixed concentration of CSF-1/IL-34-Biotin protein and serially diluted BC006 antibody were added. Additionally, CSF-1/IL-34-His protein was used as the positive control, and dilution buffer as the negative control. HRP-labeled streptavidin (SA) was used as the secondary antibody, and the amount of antigen–antibody binding was detected by enzyme-substrate color development. A four-parameter curve was fitted with the logarithm of antibody concentration as the abscissa and the OD450 value (reflecting the amount of antigen–antibody binding) as the ordinate. (2) Reporter Gene Assay. In a CHO-K1 transgenic cell line (Merck, Darmstadt, Germany) expressing human CSF-1R and luciferase responsive to SRE signals, CSF-1 could specifically activate luciferase expression in a dose-dependent manner. SRE is a commercial reporter vector pGL4.33 [luc2P/SRE/Hygro] (Promega, Madison, WI, USA). When BC006 was added, it inhibited CSF-1-induced luciferase expression mediated by downstream SRE signal transduction, and the relative light unit (RLU) value of the fluorescence signal was negatively correlated with the antibody concentration. A four-parameter curve was fitted with the logarithm of antibody concentration as the abscissa and the RLU value at the corresponding concentration as the ordinate. The IC50 values of the sample and reference standard were obtained, and the relative cellular activity of the sample was calculated.

Induction of M2 Macrophages: Peripheral blood mononuclear cells (PBMCs) were bought from AllCells (Alameda, CA, USA); they were isolated from human peripheral blood, and monocytes were further separated from PBMCs. CSF-1 was added to induce the differentiation of monocytes into macrophages. Flow cytometry (Beckman Coulter, Indianapolis, IN, USA) was used to detect the expression of CSF-1R on the cell surface to confirm the induction and differentiation efficiency. During the induction and differentiation process, serially diluted BC006 was added simultaneously. After 7 days of induction, Cell-Titer Glo detection reagent was added to determine the number of viable cells, and the cell viability relative to the antibody-untreated control group was calculated.

ADCC and CDC: The ADCC activity was evaluated using the classic PBMC-based assay. Briefly, PBMCs (AllCells, Alameda, CA, USA) were used as effector cells, and HEK293 cells overexpressing human CSF-1R served as target cells. Target cells were incubated with serial dilutions of BC006 samples, followed by the addition of effector cells. After co-incubation, LDH detection reagent (Dojindo Laboratories, Shanghai, China) was added to measure the activity of LDH released from dead target cells. The antibody-mediated cell lysis rate was calculated based on the OD values obtained from the microplate reader. A four-parameter logistic model was used to determine the relative ADCC activity (compared with the reference standard) and the maximum lysis of each sample. HEK293 cells expressing human CSF-1R were used as target cells and incubated with serially diluted samples. Rituximab (Roche, Basel, Switzerland) was set as the positive control, with CD20-expressing Raji cells serving as the corresponding target cells for the positive control. After co-incubation with human serum complement for 3 h, Cell-Titer Glo reagent was added. The relative light unit (RLU) value was measured by a microplate reader, and the relative cell lysis ratio was calculated according to the RLU readings.

IPF Organoid-on-a-chip Model: The TGF-β1-induced IPF lung organoid-on-a-chip model was constructed by Jiangsu Advant Biotechnology Co., Ltd. A high-throughput membrane chip (Advant Biotech, Suzhou, China) was used, with lung organoids (Advant Biotech, Suzhou, China) in the upper layer and endothelial cells (Meisen Biotech, Hangzhou, China) plus macrophages (Serumwerk Bernburg: Bernburg, Germany) in the lower layer. First, a normal lung chip model was constructed, and then TGF-β1 was used to induce pulmonary fibrosis to establish the IPF-induced model. The following experiments performed: immunofluorescence to detect the expression of AQP5, SFTPC, α-SMA, Collagen I, E-Cadherin, and VE-Cadherin; trans-epithelial electrical resistance (TEER) measurement (WPI, Sarasota, FL, USA); FITC-Dextran (MCE, Suzhou, China) to detect barrier permeability. A rocking perfusion system (Advant Biotech, Suzhou, China) was used to culture the organoid-on-a-chip. High-content analysis (Advant Biotech, Suzhou, China) and trans-endothelial electrical resistance measurement were used for imaging and analysis.

IPF Mouse Model: The experiment was approved by the Institutional Animal Care and Use Committee (IACUC) of Pengli Biomedical Technology Co., Ltd. (Shanghai, China), (Approval No.: PL23-0709-1; approval date: 15 April 2023), an AAALAC International-accredited facility. Mice were housed in clear polycarbonate cages (265 mm × 160 mm × 127 mm; 2–5 animals/cage) with autoclaved corn cob bedding (changed every 7–10 days). The housing environment was controlled at 20–26 °C, 40–70% humidity, 12 h light/dark cycle (08:00–20:00 light), and 15–25 air changes/hour. Animals had ad libitum access to irradiated rodent chow from Jiangsu Xietong (Nanjing, China) and RO-purified or autoclaved water, with quality records maintained. Known contaminants in feed and water were not expected to interfere with the study objectives. Mice were monitored daily for health, behavior, and signs of distress. Humane endpoints were defined as ≥20% body weight loss, persistent lethargy, anorexia, inability to access food/water, labored respiration, or moribund status. Analgesics or anesthetics were administered whenever potentially painful procedures were performed. Animals meeting these criteria were euthanized immediately. No animals died spontaneously before reaching endpoints and no animals reached the humane endpoint criteria. The total duration of the study was 28 days. All animals were humanely euthanized in strict accordance with the IACUC. Euthanasia was performed via gradual CO_2_ inhalation followed by cervical dislocation, consistent with AVMA guidelines. All research personnel involved in animal husbandry and experimental procedures had received specialized professional training in animal handling, care, and ethics to ensure the highest standards of welfare. Animals were administered bleomycin (Nippon Kayaku, Tokyo, Japan) via the intratracheal route at a dose of 2 U/kg with an administration volume of 2.5 mL/kg. The administration started on Day 5 and continued until the end of the experiment on Day 21.

In this study, 53 female B-hCSF-1&CSF-1R transgenic mice (C57BL/6 background) and 5 female wild-type C57BL/6 mice were utilized. All animals were housed under specific pathogen-free (SPF) conditions; they were aged 6–8 weeks, with a mean body weight of 20 ± 0.2 g. A total of 48 transgenic mice and 3 wild-type C57BL/6 mice were screened based on body weight and randomly assigned to experimental groups using simple random sampling via the Bioknow RTSM software (version 3.1, Bioknow Info-Tech, Beijing, China). All animal inclusion, exclusion, and data screening criteria were defined a priori. Animals with normal physical condition and qualified age/body weight were enrolled. Full double-blinding was implemented throughout the study. An independent statistician retained the sealed randomization list, while all operators, outcome assessors, and data analysts remained blinded to group assignments until all experimental data were locked. The normal group was designed only as a baseline control for systematic monitoring and model validation. Given the minimal inter-individual variation in wild-type mice, only 3 animals were allocated to this group. All remaining experimental groups consisted of 12 animals per group, which fully meets the statistical requirements for this study.

B-hCSF1/CSF1R transgenic mice and C57BL/6 mice were purchased from Jiangsu Biocytogen (Nantong, China). A pulmonary fibrosis model was established in B-hCSF1/CSF1R transgenic mice by intratracheal administration of bleomycin. Pulmonary function testing (PFT) was performed on enrolled animals using a pulmonary function analyzer, employing indicators such as the pressure–volume (PV) curve, forced vital capacity (FVC), inspiratory capacity (IC), vital capacity (VC), dynamic lung compliance (Cdyn), and chord compliance (Cchord). Wright–Giemsa staining solution was used to stain different immune cells, and a total number of 200 cells underwent differential counting under an optical microscope (Nikon, Tokyo, Japan). Mouse lung samples were fixed and made into paraffin sections. The entire section was observed from left to right; Masson staining was used for fibrosis evaluation, F4/80 immunohistochemical staining and section quantification were conducted, and CD206 immunohistochemical staining (Sino Biological, Beijing, China) and section quantification were performed to evaluate relevant indicators.

Cynomolgus Monkey Safety Evaluation: This study was performed in accordance with the National Standard of the People’s Republic of China GB 14925-2010 and approved by the Institutional Animal Care and Use Committee (IACUC) of Huace Biotechnology Co., Ltd. (Suzhou, China). (Approval No.: IACUC-A2019042-T005-01; approval date: (19 October 2020), an AAALAC International-accredited facility. Cynomolgus monkeys were individually housed in stainless steel cages (80 cm × 90 cm × 100 cm) in a controlled environment: temperature 19.2–23.1 °C, relative humidity 50.8–88.7%, 12 h light/dark cycle, and ≥8 air changes per hour. Animals were provided with commercial monkey maintenance chow (Keao Xieli Feed, Beijing, China) twice daily, fresh fruits or vegetables approximately 50 g at least 3 times weekly, and reverse-osmosis water ad libitum. Environmental enrichment was provided in the form of toys. Animals were monitored twice daily by trained animal care staff for general health, clinical signs, behavior, appetite, and water intake. Detailed clinical observations included appearance, fur, eyes, respiration, posture, activity, and signs of pain or distress. Body weight was measured periodically, and health status was assessed using criteria including body weight loss, decreased activity, abnormal respiration, reduced food or water intake, and lethargy. Humane endpoints were defined as ≥20% body weight loss, persistent lethargy, anorexia, inability to access food/water, labored respiration, or moribund status. Analgesics or anesthetics were administered whenever potentially painful procedures were performed. Animals meeting these criteria were euthanized immediately. No animals died spontaneously before reaching endpoints and no animals reached the humane endpoint criteria. The total duration of the study was 42 days. All animals were humanely euthanized in strict accordance with the IACUC. Prior to euthanasia, the animals were fasted for at least 8 h. Anesthesia was induced via an intravenous injection of sodium pentobarbital (Sinopharm Chemical Reagent, Beijing, China) at a dose of 30 mg/kg (concentration: 20 mg/mL; injection volume: 1.5 mL/kg body weight). All research personnel involved in animal husbandry and experimental procedures had received specialized professional training in non-human primate handling, care, and ethics to ensure the highest standards of welfare.

On the premise of meeting research objectives and scientific criteria, the minimum feasible number of animals was adopted. A total of 6 cynomolgus macaques were used in this trial, which satisfies the minimum animal quantity requirement for generating sufficient experimental data. Healthy animals were selected as test subjects; female animals were required to be nulliparous and non-pregnant. All macaques were of conventional grade and confirmed five-pathogen-negative. At randomization, male cynomolgus macaques weighed 2.41–2.56 kg, and females weighed 2.32–2.51 kg. The age of all animals ranged from 3.1 to 3.2 years at grouping. Prior to dosing, all monkeys scheduled for the trial underwent two rounds of examinations including general clinical signs, body weight, body temperature, respiration, lead II electrocardiogram, blood pressure, hematology, coagulation, serum biochemistry and urinalysis, as well as one ophthalmological examination. Animals meeting all eligibility criteria were enrolled in the study. The six cynomolgus macaques were stratified by sex and randomly allocated to three groups via simple random sampling generated by Bioknow RTSM software (version 3.1, Bioknow Info-Tech, Beijing, China), namely, vehicle control group, BC006 low-dose group, and BC006 high-dose group, with 2 animals per group (1 male and 1 female per group). Full double-blinding was implemented throughout the trial: an independent statistician retained the sealed randomization list, and all animal operators, outcome assessors, and data analysts remained blinded to group assignments until all data were locked and the database finalized. The allocation of 2 cynomolgus macaques per group aligns with international acute toxicity test guidelines and the 3R principle. Acute toxicity evaluations rely primarily on qualitative adverse clinical observations and do not require large animal cohorts; the three-group design enables the reliable detection of test article-related adverse reactions. For acute toxicity assessment, the low and high intravenous doses of BC006 were set at 203 mg/kg and 406 mg/kg, respectively. The injection volume was 20 mL/kg via intravenous administration. Each animal received a single dose within 24 h, and all animals were continuously monitored for 42 days post-dosing. 

Statistical Analysis: SPSS 26 or GraphPad Prism 10 was used for data analysis. Experimental data were expressed as mean ± SEM. Box plots were used for outlier identification; after excluding abnormal outliers, statistical analysis was performed. A *p*-value < 0.05 was considered statistically significant.

## 3. Results

### 3.1. Antibody Screening, Binding Activity, and Affinity

Using the phage Fab display library DPL6, three rounds of screening were performed, and monoclonal colonies specifically binding to the human CSF-1R extracellular domain were identified by ELISA. DNA sequencing and sequence alignment of the ELISA-positive clones yielded 39 unique antibody Fab sequences, among which 15 unique sequences showed significant enrichment. These Fab fragments were further purified, followed by characterization of physicochemical properties and amino acid sequence analysis via SDS-PAGE and ForteBio. Finally, clone 7204 was selected and reformatted into fully human IgG1 as the anti-CSF-1R antibody (BC006).

The binding activity between BC006 and CSF-1R was detected by ELISA. Results showed that BC006 could specifically bind to human CSF-1R, with a mean EC50 of 226 ± 57 nM. Using the ForteBio platform based on BLI technology, results demonstrated that the mean binding affinity (KD) of BC006 for CSF-1R was 30 ± 2 nM, indicating excellent binding activity.

### 3.2. CSF-1R Binding Domain of BC006

**Figure 1:**
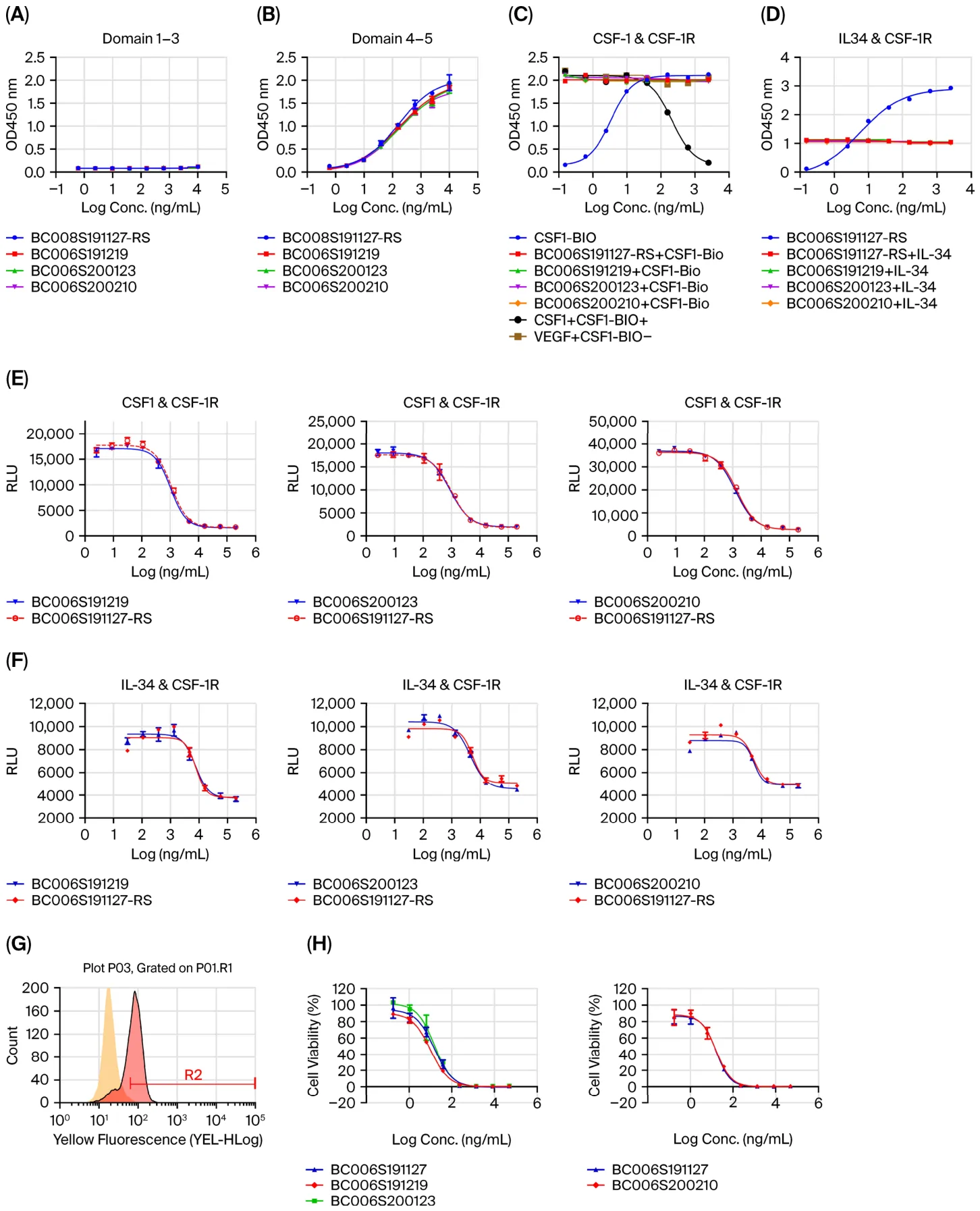
BC006 binding domain and functional efficacy. (**A**) Binding of BC006 to the human CSF-1R Ig1-3 domain. BC006S191127 represents the reference product, while BC006S191219, BC006S200123, and BC006S200210 are samples of different batches of the CSF-1R antibody. (**B**) Binding of BC006 to the human CSF-1R Ig4-5 domain. (**C**) Effect of BC006 on the CSF 1 and CSF 1R interaction determined by ELISA. (**D**) Effect of BC006 on the IL-34 and CSF 1R interaction determined by ELISA. (**E**) Effect of BC006 on the CSF-1 and CSF-1R interaction determined by reporter gene assay. (**F**) Effect of BC006 on the IL-34 and CSF-1R interaction determined by reporter gene assay. (**G**) Flow cytometric results of CSF-1R expression on the macrophage surface. (**H**) BC006 inhibits the induced differentiation of monocytes into M2-type macrophages in a dose-dependent manner.

The extracellular domain of human CSF-1R consists of five domains (Ig1–Ig5). Among them, domains 1–3 (Ig1–Ig3) are involved in binding to the ligand CSF-1 and IL-34, while domains 4–5 (Ig4–Ig5) are associated with receptor dimerization. Under static conditions, CSF-1R exists as disordered monomers on the cell membrane. When its Ig2 and Ig3 domains bind to the dimeric ligand, adjacent CSF-1R molecules are recruited to form a tetrameric complex. At this point, the Ig4 and Ig5 regions of the two CSF-1R molecules are brought close to each other to form a dimer. To determine the binding domain of BC006 on CSF-1R, the ELISA method was used to detect the binding activity of BC006 to different domains of human CSF-1R. The results showed that BC006 only bound to the Ig4–Ig5 domains with an EC50 of 1193 ± 180 nM and did not bind to the Ig1–Ig3 domains (Figure 1A,B). Therefore, BC006 blocks the signal transduction after CSF-1 binding by interacting with the Ig4–Ig5 domains of CSF-1R, without directly affecting the interaction between ligand and CSF-1R.

### 3.3. BC006 Inhibits Signaling Without Blocking Ligand Binding

CSF-1 and IL-34 are ligands of CSF-1R. The binding of ligands to the receptor can initiate the downstream signaling pathway. Theoretically, BC006, a product targeting CSF-1R, can bind to CSF-1R and block signal transduction, thereby affecting macrophage polarization. Here, ELISA and reporter gene assays were used to detect the effect of BC006 on the interactions between CSF-1 and CSF-1R and between IL-34 and CSF-1R. A competitive ELISA showed that BC006 had no effect on the interactions of CSF-1/CSF-1R and IL-34/CSF-1R at the protein level (Figure 1C,D). In the reporter gene assay, BC006 inhibited SRE signal transduction at the cellular level and affected the interactions between CSF-1 and CSF-1R and between IL-34 and CSF-1R. The IC50 values were 7093 ± 1100 nM and 40,469 ± 11,000 nM, respectively. The affected reporter gene activities ranged from 107% to 116% and 98% to 119%, respectively (Figure 1E,F). In summary, BC006 does not directly block the CSF-1 and CSF-1R or IL-34 and CSF-1R interaction but instead inhibits downstream signaling by binding to the Ig4-5 domains of CSF-1R.

### 3.4. BC006 Inhibits Monocyte Differentiation into M2-Type Macrophages

Macrophages are mainly divided into M1 and M2 subtypes. M1 macrophages are characterized by pro-inflammatory cytokine secretion and strong microbicidal activity, while M2 macrophages play a central role in promoting tissue repair and are the major mediators of pathological fibrosis. CSF-1 can induce the differentiation of human monocytes into M2-type macrophages, thereby affecting the immune microenvironment and promoting fibrosis.

Peripheral blood mononuclear cells (PBMCs) were isolated from human peripheral blood, and monocytes were further separated from PBMCs. Then, CSF-1 was added to induce the differentiation of monocytes into M2-type macrophages. CSF-1R was expressed on 67% of the macrophage surface, confirming the successful induction of M2-type macrophages (Figure 1G). During the induction and differentiation process, gradient diluted BC006 was added simultaneously. After 7 days of induction, we used Cell-Titer Glo (CTG) to detect the number of viable cells, which showed that IC50 was 101 ± 19 nM. BC006 could inhibit the induced differentiation of monocytes into M2-type macrophages in a dose-dependent manner (Figure 1H).

### 3.5. In Vitro Safety Evaluation of BC006

**Figure 2:**
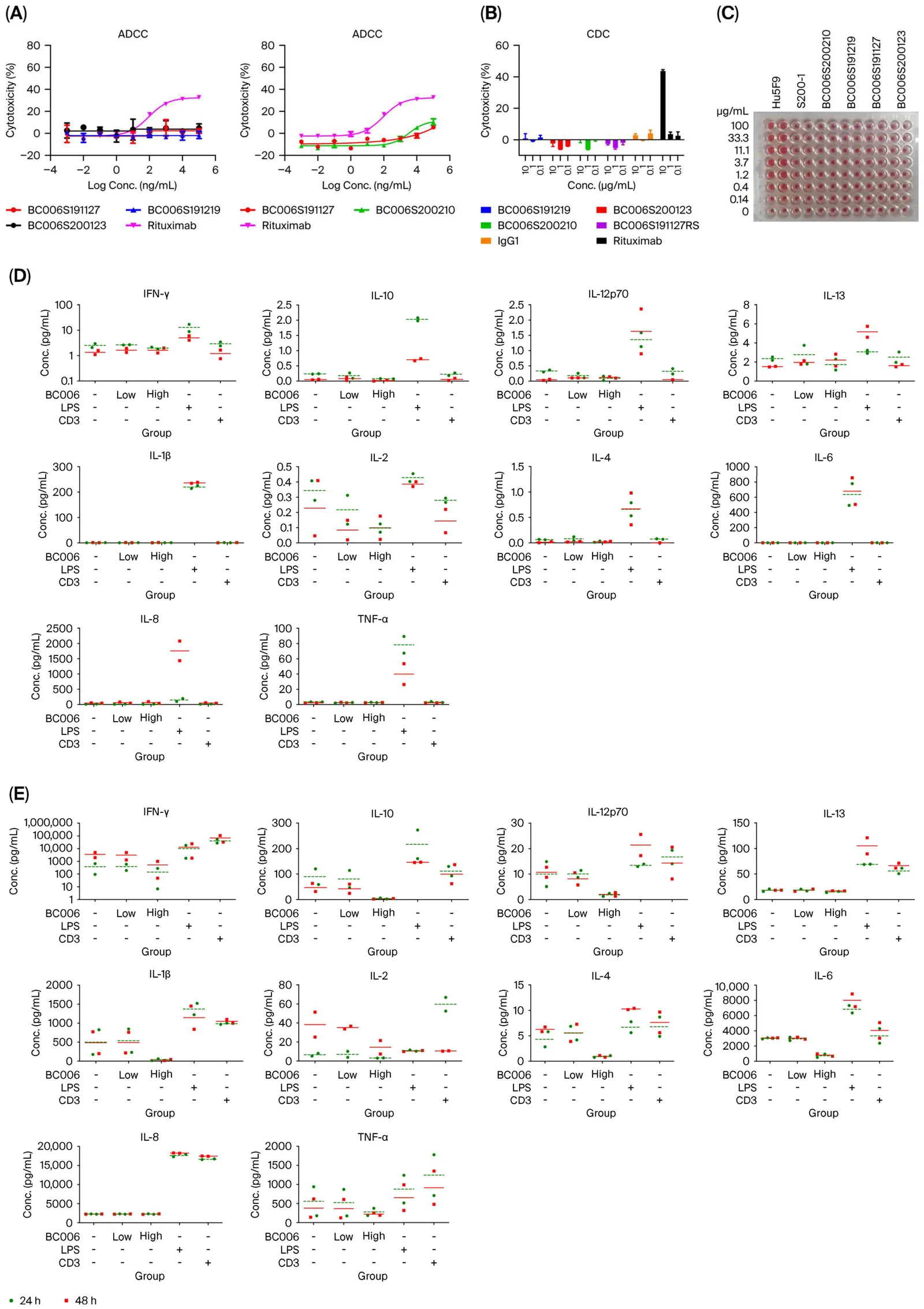
In vitro safety evaluation of BC006. (**A**) ADCC activity of BC006. (**B**) CDC activity of BC006. (**C**) Hemagglutination assay of human erythrocytes incubated with BC006. (**D**) Expression levels of cytokines secreted from human whole blood stimulated by BC006. The high and low concentrations were 250 μg/mL and 0.25 μg/mL, respectively. (**E**) Expression levels of cytokines secreted from human PBMCs stimulated by BC006.

BC006 exerts its anti-fibrotic effects by just blocking the CSF-1 signaling pathway. It does not require the antibody-dependent cellular cytotoxicity (ADCC) and complement-dependent cytotoxicity (CDC) activities which conventional antibodies typically possess. Moreover, ADCC and CDC activities may conversely introduce potential safety risks. In contrast to the control group, BC006 showed no obvious dose-dependent ADCC or CDC activities (Figure 2A,B).

Antibodies may form antigen–antibody complexes with specific antigens on the surface of erythrocytes, leading to hemagglutination and subsequent lysis in humans, thereby causing seriously adverse reactions. Therefore, it is necessary to evaluate the potential risk of BC006 to induce hemagglutination in vitro. In contrast to the positive control group Hu5F9 (anti-CD47 antibody), no agglutination was observed after incubating erythrocytes with BC006 samples of different concentrations and batches for 3 h (Figure 2C).

To evaluate the potential risk of BC006 to induce cytokine release syndrome, the effects of BC006 on the release of ten cytokines, namely, IFN-γ, IL-10, IL-12p70, IL-13, IL-1β, IL-2, IL-4, IL-6, IL-8, and TNF-α, from human whole blood and PBMCs were assessed using electrochemiluminescence technology based on the MSD platform. Compared with the control groups, BC006 at both low and high concentrations exerted no significant effects on the release of the above-mentioned cytokines in human whole blood or PBMCs, indicating a low risk of cytokine release syndrome induced by BC006 in preclinical studies (Figure 2D,E).

In conclusion, BC006 exhibits no ADCC or CDC effects, does not induce cytokine release from PBMCs or whole blood, and does not cause hemagglutination, demonstrating a favorable in vitro safety profile.

### 3.6. In Vitro Efficacy of BC006 in Induced IPF Organoid-on-a-Chip Model

**Figure 3:**
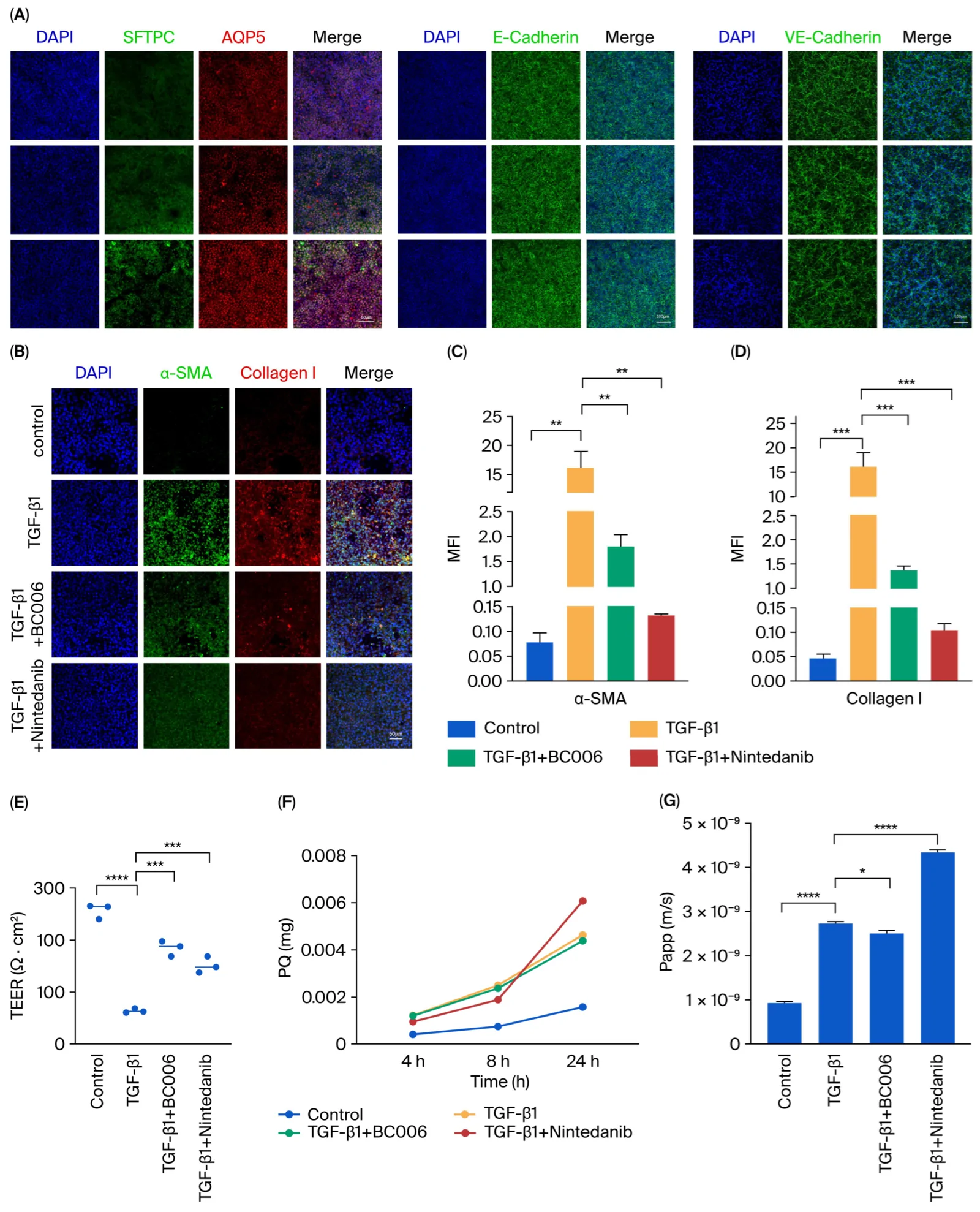
In vitro efficacy of BC006 in induced IPF organoid-on-a-chip model. (**A**) Immunofluorescence staining of the normal lung organoid chip model, including the expression of type I and type II alveolar cell markers (AQP5 and SFTPC), as well as the cell tight junction markers E-Cadherin and VE-Cadherin. (**B**) Immunofluorescence staining of α-SMA and Collagen I expression (*n* = 3; 25 ng/mL TGF-β; 1000 µg/mL BC006; 10 µM Nintedanib). (**C**) Mean fluorescence intensity (MFI) of α-SMA using immunofluorescence. (**D**) MFI immunofluorescence tests of Collagen I. (**E**) Measurement of TEER value using a resistance meter after administration. (**F**) Detection of penetration quality (PQ) using immunofluorescence. (**G**) Apparent Permeability Coefficient (Papp), Papp = (dQ/dt)/(A × C0). (* *p* < 0.05, ** *p* < 0.01, *** *p* < 0.001, **** *p* < 0.0001).

A high-throughput organoid-on-a-chip was used to construct a normal lung chip model. The upper layer of the chip contained lung organoids and macrophages, while the lower layer contained endothelial cells. In the normal lung organoid-on-a-chip model, the marker of type I alveolar cells (AQP5) and the marker of type II alveolar cells (SFTPC) were both expressed. The epithelial cell junction protein marker (E-Cadherin) and HUVEC endothelial cell junction protein (VE-Cadherin) were highly expressed. The trans-epithelial electrical resistance (TEER) value was above 300 Ω·cm^2^, and the permeability coefficient remained nearly close. These results indicated that the normal lung organoids and endothelial cells were in good growth status, with tight intercellular junctions and intact model barrier function, confirming the successful construction of the normal lung organoid-on-a-chip model (Figure 3A). 

Fibrosis was induced with 25 ng/mL TGF-β1 on Day 12. In the TGF-β1-induced IPF organoid-on-a-chip model, the cells became elongated and shrunken, intercellular gaps emerged, tight junctions were damaged, the expression of Collagen I and α-SMA increased significantly, the TEER value decreased remarkably, and the barrier integrity was disrupted (Figure 3B–E). After treating BC006 and Nintedanib for 48 h, the immunofluorescence results revealed that the TGF-β1-induced group without drug treatment had high Collagen I and α-SMA expression, while the BC006 group and Nintedanib group showed a significant decrease in the expression of Collagen I and α-SMA (Figure 3C,D). The TEER value and barrier permeability showed that BC006 and Nintedanib could repair the barrier integrity damaged by TGF-β1 and promote the formation of tight intercellular junctions (Figure 3E–G). By inhibiting the CSF1-CSF1R signaling pathway, reducing fibrin expression and chemokine secretion, and maintaining the barrier function of lung tissue, BC006 exerts a certain anti-fibrotic effect in the induced IPF organoid-on-a-chip model. 

### 3.7. In Vivo Therapeutic Efficacy of BC006 in an IPF Mouse Model

A mouse model of pulmonary fibrosis was induced by intratracheal administration of bleomycin. Subsequently, the successfully induced pulmonary fibrosis mice were treated with CSF-1R antibody by intravenous injection (i.v.) to evaluate the antibody’s inhibitory effect on M2 macrophages and its efficacy in treating IPF. Compared with mice in the normal group, B-hCSF-1/CSF-1R transgenic mice that received intratracheal administration of bleomycin exhibited typical pathological features of IPF. These features included fibrotic pathological changes in lung tissue, increased leukocytes in bronchoalveolar lavage fluid, elevated lung tissue inflammation scores and fibrosis percentage, and impaired lung function. These results confirm the successful establishment of the bleomycin-induced pulmonary fibrosis model.

**Figure 4:**
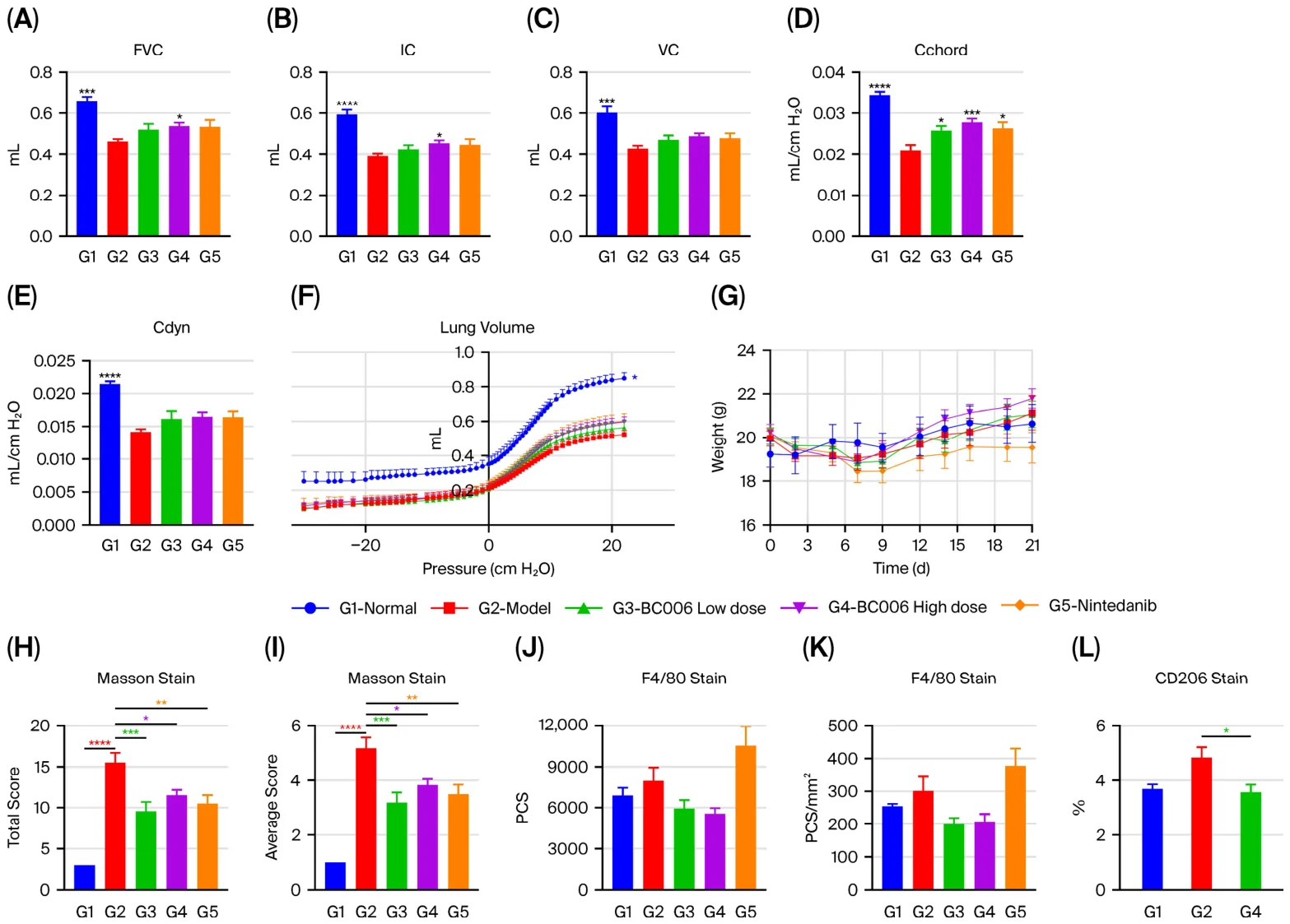
In vivo therapeutic efficacy of BC006 in an IPF mouse model. (**A**) Detection of forced vital capacity (FVC) using a pulmonary function analyzer. G1: *n* = 3; G2-G5: *n* = 12. (**B**) Inspiratory capacity (IC). (**C**) Vital capacity (VC). (**D**) Chord compliance (Cchord). (**E**) Dynamic lung compliance (Cdyn). (**F**) Pressure–volume (PV) curve. (**G**) Changes in body weight growth rate before and after treatment. (**H**) Masson staining for fibrosis evaluation (total score). (**I**) Masson staining for fibrosis evaluation (average score). (**J**) F4/80 immunohistochemical staining for total positive cells (PCS). (**K**) F4/80 immunohistochemical staining for total positive cell density. (**L**) CD206 immunohistochemical staining for proportion of positive cells; % = CD206 positive cells/total cells. (* *p* < 0.05, ** *p* < 0.01, *** *p* < 0.001, **** *p* < 0.0001).

Pulmonary function tests (PFTs) were performed in mice using a pulmonary function analyzer, including measurements of vital capacity (VC), forced vital capacity (FVC), inspiratory capacity (IC), pressure–volume (PV) curve, dynamic lung compliance (Cdyn), and chord compliance (Cchord). Compared with the normal group, all lung function indicators in the model group decreased significantly. All drug-administered groups showed a trend of improving lung function. However, the high-dose BC006 group could significantly improve the lung function parameters FVC (*p* < 0.05), IC (*p* < 0.05), and Cchord (*p* < 0.005); the low-dose BC006 group also showed a significant improvement in the lung compliance indicator Cchord (*p* < 0.05) (Figure 4A,B,D). Compared with the induced group, the VC and Cdyn parameters in all treatment groups showed an upward trend, but none of the increases reached statistical significance (Figure 4C,E). The PV curve of the high-dose group was essentially consistent with that of the Nintedanib group (Figure 4F). Overall, the improvement of lung function indicators in the BC006 group was slightly better than that in the Nintedanib group. The body weight of mice increased in all groups except for the Nintedanib group, which indicates that Nintedanib has certain toxicity in the mouse model, and BC006 has better safety (Figure 4G). Fibrosis scoring indexes showed that the model group had multifocal aggregation, fibrotic thickening, and a significant increase in fibrosis score. Total and average score of Masson staining indicated that the fibrosis score of the induced group increased significantly whereas those of the drug treatment groups were all significantly decreased (Figure 4H,I). For F4/80 staining, the total number of positive cells and total positive cell density increased in the control and Nintedanib group, but these showed a decreasing trend in the BC006 group (Figure 4J,K). For CD206 staining, the value in the BC006 group decreased significantly compared with the model group. This means that the M2 macrophage level in the BC006 group decreased significantly (Figure 4L). These results suggest that BC006 exerts anti-IPF efficacy by regulating the proliferation of mature macrophages and M2 polarization, and its regulatory mechanism may differ from that of Nintedanib.

Compared with the model group, the positive drug Nintedanib significantly reduced detection indicators such as inflammatory cell infiltration in lung tissue, fibrosis score, and fibrosis area measurement, exhibiting a good effect on improving fibrosis. BC006 reduced the number of pulmonary inflammatory cells, improved lung function, decreased the fibrosis score, and reduced the fibrosis area. It can significantly reduce lung tissue damage and inflammatory cell infiltration caused by pulmonary fibrosis, thereby improving lung function and suggesting a good therapeutic effect on the disease. Further immunohistochemical detection of M2 macrophages in tissue samples revealed that BC006 had a significant inhibitory effect on M2 macrophages. This suggests that BC006 inhibits overactivated M2-type macrophages, thereby regulating macrophage balance and reducing fibrotic-like tissue changes caused by excessive tissue repair involving macrophages. In summary, the BC006 antibody has a good effect on reducing inflammation and alleviating fibrosis, and this effect is mainly achieved by regulating the overactivation of M2 macrophages.

### 3.8. In Vivo Safety Evaluation of BC006 in Cynomolgus Monkeys

**Figure 5:**
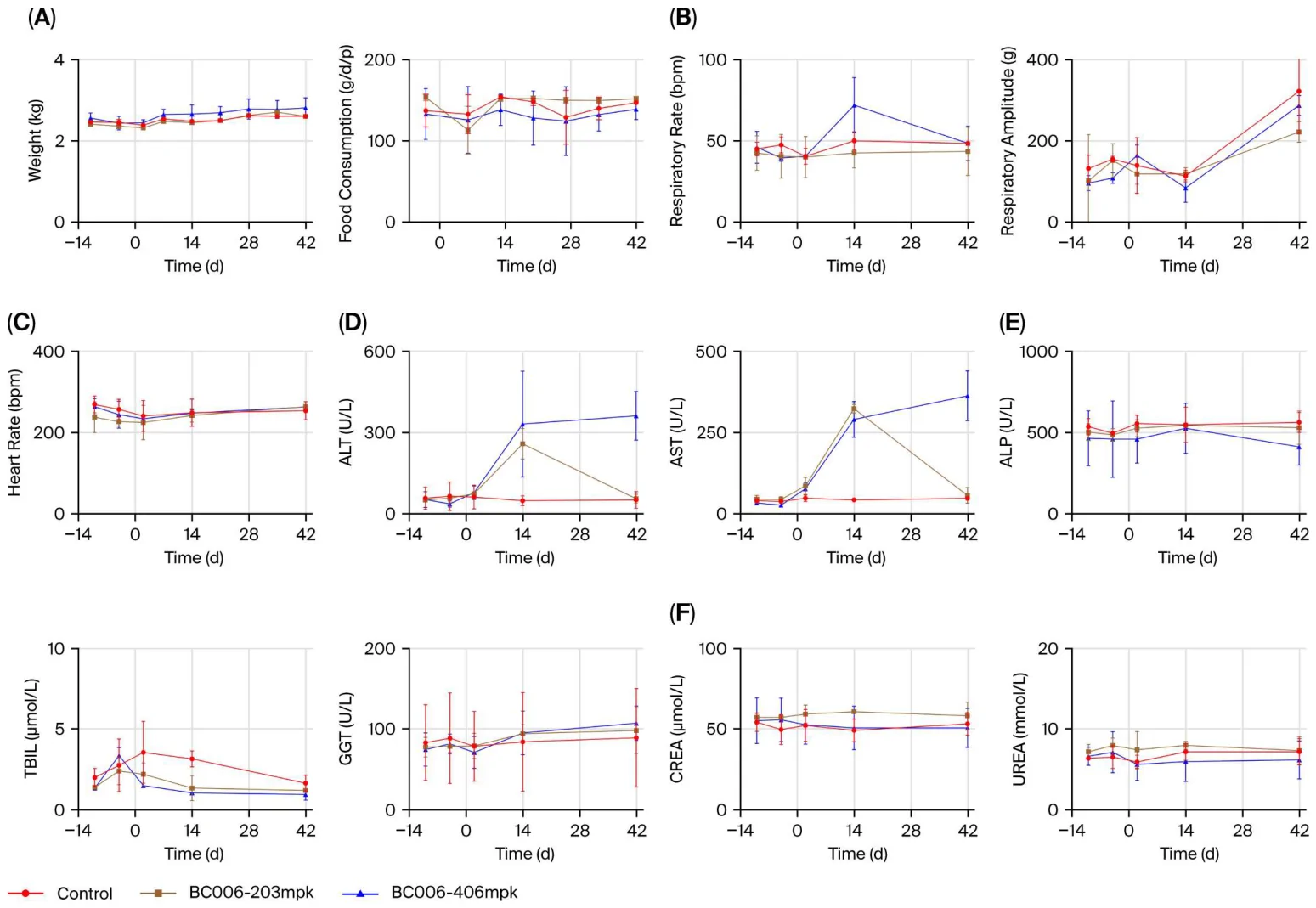
In vivo safety evaluation of BC006 in cynomolgus monkeys. (**A**) Body weight and food consumption; *n* = 2. (**B**) Respiratory rate and respiratory amplitude. (**C**) Heart rate. (**D**) Aspartate aminotransferase (AST) and alanine aminotransferase (ALT). (**E**) Alkaline phosphatase (ALP), total bilirubin (TBIL), and gamma-glutamyl transferase (GGT). (**F**) Creatinine (CREA) and blood urea nitrogen (UREA).

No obvious abnormalities were noted in body weight or food consumption in the treatment groups (Figure 5A). Respiratory rate and respiratory amplitude remained unremarkable (Figure 5B). Heart rate did not change much during the experiment time (Figure 5C). Serum biochemistry parameters were within normal ranges. In the low-dose group, AST and ALT levels increased by 5–6 times and returned to normal during the recovering period. In contrast, the AST and ALT levels rose by 5–7 times and showed no signs of recovery after 6 weeks, with a transient additional increase observed (Figure 5D). Subsequent pathological sections revealed that the transient elevations of ALT and AST were pharmacological amplification and secondary effects, which did not result in any hepatic injury even at a high dose. Cholestasis and bilirubin metabolism showed no significant change based on ALP, TBIL, and GGT levels (Figure 5E). This indicates that the biliary tract is unobstructed, with no significant cholestatic injury to hepatocytes and no obvious inflammation or destruction of the biliary epithelium. Renal filtration and excretory function were not affected by the CREA and UREA results (Figure 5F). Although the normal dose of BC006 is 3 mg/kg in clinical trials, we needed to use an extraordinarily high dose to evaluate toxicity in cynomolgus monkeys. All the safety parameters except AST and ALT exhibited no significant change. Even at a very high dose (203 mg/kg), AST and ALT could decrease to baseline normal levels during the recovery period. These findings indicate that BC006 possesses a favorable overall safety profile compared to many small-molecular drugs.

## 4. Discussion

CSF-1R antibodies exhibit a different mechanism of action compared to other drugs targeting the same target [20]. They do not affect the interaction between CSF-1 or IL-34 and CSF-1R but exert biological functions by inhibiting downstream receptor dimerization [21]. Ligand-blocking antibodies and dimerization-blocking antibodies exhibit similar suppression of ligand-dependent PI3K/AKT, MAPK/ERK, PLCγ2 and SFK signaling. Nevertheless, only dimerization-blocking antibodies suppress ligand-independent signaling, including pre-dimerization and oncogenic mutation-driven dimerization [22]. Compared with small-molecule drugs targeting the same receptor, CSF-1R antibodies can bind to the receptor specifically and eliminate off-target toxicity. In contrast, small-molecule drugs, due to their relatively simple structure, tend to bind to proteins or receptors other than the target, leading to off-target effects and toxic side effects [23,24]. For example, Pexidartinib, a marketed small-molecule drug targeting CSF-1R, has been reported to cause various toxic side effects, particularly hepatotoxicity, including liver failure, vanishing bile duct syndrome, bile duct reduction, symptomatic cholestasis, cholangitis, and liver disease [25]. The principal advantage of BC006 lies in its better safety profile and different mechanism of action for IPF. Although BC006 showed no ADCC, CDC, hemagglutination, cytokine storms, or obvious toxic side effects (such as hepatotoxicity) in preclinical and early clinical studies, relevant toxicities need to be further monitored in later-phase clinical trials.

In this study, an induced pulmonary fibrosis organoid-on-a-chip model was constructed. The results show that BC006 exerts an inhibitory effect on idiopathic pulmonary fibrosis by inhibiting the CSF1-CSF1R signaling pathway specifically, reducing fibrin expression and chemokine secretion to maintain the barrier function of lung tissue, and decreasing the production of M2-type macrophages and the secretion of inflammatory factors, accompanied by a reduction in the fibrosis-related protein α-SMA and Collagen I. In the normal lung organoid-on-a-chip model, the detection results were like those of pulmonary organoid-on-a-chip models reported in other studies. Compared with in vitro cell experiments, organoid-on-a-chip models have better spatial structure and microfluidic flow. In this model, the markers of type I or II alveolar cells (AQP5 and SFTPC) are expressed more effectively, cells are arranged more regularly with tighter connections, and the models better simulate the real in vivo biological environment. Meanwhile, the use of human-derived cells avoids species differences observed in animal models, making organoid-on-a-chip models an emerging tool for in vitro efficacy and toxicology evaluation [26,27].

In the mouse animal model, both the Nintedanib group and BC006 group showed anti-fibrotic effects, but their mechanisms of action differed. Nintedanib is a multi-target tyrosine kinase inhibitor that exerts anti-IPF effects by targeting PDGF, VEGF, FGF, and EGFR to inhibit fibroblast proliferation and migration; acting on IPF-related signaling pathways (TGF-β and Src); and promoting fibroblast apoptosis. BC006 mainly exerts its effects by inhibiting overactivated M2-type macrophages, reducing the number of inflammatory cells, regulating macrophage balance, and decreasing fibrotic-like tissue changes caused by excessive tissue repair involving macrophages. We speculate that BC006 exerts antifibrotic activity not only by modulating macrophage polarization, but also indirectly alleviates epithelial dysfunction and fibroblast activation. Macrophages function as a central hub mediating paracrine communication in the fibrotic microenvironment. By blocking CSF-1R, it reduces macrophage-derived profibrotic mediators including PDGFA and TGF-β1, and relieves macrophage-triggered epithelial injury. This intervention disrupts the feedforward fibrotic loop among epithelial cells, macrophages and fibroblasts. Further studies are needed to confirm these mechanisms [28]. Overall, the BC006 antibody has better safety, slightly better overall efficacy indicators, and significantly better performance in three lung function indicators. For these two anti-fibrotic drugs with different mechanisms of action, further research on their combination therapy to achieve better anti-fibrotic efficacy can be conducted in the future [29,30]. Nintedanib inhibits fibroblast proliferation, and BC006 targets macrophage CSF-1R signaling. The two drugs interrupt different segments of the self-sustaining macrophage-fibroblast fibrotic circuit. Compared with the weak off-target CSF-1R suppression by nintedanib, BC006 enables robust and selective blockade of CSF-1R. Supporting this rationale, preclinical studies confirm that combined CSF-1R inhibition and nintedanib treatment exerts synergistic antifibrotic effects superior to monotherapy [31].

BC006 is predicted to provide more benefits for three IPF patient subgroups: macrophage-high endotype patients defined by CSF-1-related biomarkers, progressive patients failing standard antifibrotic treatment, and subjects with early-to-moderate disease prior to irreversible matrix deposition. Its unique mechanism enables combinatorial application with nintedanib. Further research including biomarker validation, enriched clinical trials and combination studies is necessary to define the precise clinical niche, and non-invasive macrophage imaging may facilitate pharmacodynamic assessment [32,33].

Radi investigated the relationship between the CSF-1/CSF-1R pathway and changes in macrophage and liver enzyme levels [34]. Rats were treated with Clophosome, a macrophage-depleting agent that eliminates macrophages in various tissues containing hepatic Kupffer cells. Significant elevations in ALT and AST were observed after treatment. Similarly, marked increases in these liver enzymes were also found in CSF-1-deficient mice. These findings suggest that the elevated ALT and AST may result from reduced hepatic clearance of these enzymes secondary to the depletion of Kupffer cells. This effect can be regarded as an extension of the pharmacological action of anti-CSF-1R agents [35,36] and is a known side effect of CSF-1 and CSF-1R pathway blockade. Encouragingly, no hepatotoxicity consistent with Hy’s Law has been identified in 84 patients enrolled in the ongoing clinical trial for tenosynovial giant cell tumor. Immunogenicity is an important risk for therapeutic antibodies. As a fully human antibody, BC006 possesses low immunogenicity. Clinical data showed an ADA incidence of less than 2%, which is markedly lower than the common rate for therapeutic antibodies [37]. Although BC006 induces a transient elevation of AST and ALT, which rapidly return to normal levels, its overall safety profile is still superior to that of marketed small-molecule drugs for IPF treatment. The approved anti-CSF-1R antibody axatilimab establishes precedent for chronic antibody administration, though special safety surveillance should be implemented for vulnerable IPF patient populations. Given the chronic progressive characteristics of IPF, the short-term observation window of current preclinical studies imposes limitations. We intend to perform 13-week and 26-week chronic toxicity tests following ICH S6(R1), and adopt prolonged bleomycin fibrosis models to verify sustained efficacy [38]. 

## Data Availability

The datasets generated and analyzed during the current study are available from the corresponding author on reasonable request.

## Abbreviations

The following abbreviations are used in this manuscript:

ADCC	Antibody-dependent cellular cytotoxicity
ALP	Alkaline phosphatase
ALT	Alanine aminotransferase
AQP5	Aquaporin 5
AST	Aspartate aminotransferase
α-SMA	Alpha-smooth muscle actin
CDC	Complement-dependent cytotoxicity
Cdyn	Dynamic compliance
Cchord	Chord compliance
CHO-K1	Chinese hamster ovary-K1
CREA	Creatinine
CSF-1	Colony-stimulating factor 1
CSF-1R	Colony-stimulating factor 1 receptor
CTGF	Connective tissue growth factor
DAPI	4′,6-diamidino-2-phenylindole
EC50	Half maximal effective concentration
ECM	Extracellular matrix
EGFR	Epidermal growth factor receptor
ELISA	Enzyme-linked immunosorbent assay
FGF	Fibroblast growth factor
FVC	Forced vital capacity
GGT	Gamma-glutamyl transferase
IC	Inspiratory capacity
IC50	Half maximal inhibitory concentration
IFN-γ	Interferon-gamma
IgG	Immunoglobulin G
IL	Interleukin
IPF	Idiopathic pulmonary fibrosis
i.v.	Intravenous
JAK-STAT	Janus kinase–signal transducer and activator of transcription
KD	Equilibrium dissociation constant
LPA	Lysophosphatidic acid
M1/2	M1/M2 macrophage
nM	Nanomolar
OD	Optical density
Papp	Apparent permeability coefficient
PBMCs	Peripheral blood mononuclear cells
PDGF	Platelet-derived growth factor
PDE4	Phosphodiesterase 4
PFT	Pulmonary function test
PTX-2	Pentraxin 2
RLU	Relative light unit
SDS-PAGE	Sodium dodecyl sulfate-polyacrylamide gel electrophoresis
SFTPC	Surfactant protein C
TBIL	Total bilirubin
TEER	Trans-epithelial electrical resistance
TGF-β1	Transforming growth factor-beta 1
TKI	Tyrosine kinase inhibitor
TNF-α	Tumor necrosis factor-alpha
VC	Vital capacity
VE-Cadherin	Vascular endothelial cadherin
VEGF	Vascular endothelial growth factor
